# Constitutional symptoms and response to Penicillin G in erysipelas and cellulitis – a monocentric, retrospective, explorative study

**DOI:** 10.1111/ddg.15957

**Published:** 2026-01-21

**Authors:** Helena Schieffers, Cord Sunderkötter

**Affiliations:** ^1^ Department of Dermatology and Venereology University Hospital Halle Martin Luther University Halle‐Wittenberg Halle (Saale) Germany; ^2^ MSB Medical School Berlin University of Health and Medicine Berlin Germany

**Keywords:** Cellulitis, constitutional symptoms, erysipelas, penicillin, skin and soft tissue infections

## Abstract

**Background**: Erysipelas, caused by streptococci, should be treated with penicillin, while uncomplicated cellulitis (phlegmon), often caused by *Staphylococcus aureus*, requires penicillinase‐resistant beta‐lactam antibiotics, which have a higher risk of adverse effects. Distinguishing between these infections is important. Constitutional symptoms like chills and fever may help in diagnosis. Therefore, we have compared retrospectively how frequently patients with erysipelas versus patients with cellulitis have experienced constitutional symptoms and whether they responded to penicillin.

**Patients and Methods**: We retrospectively evaluated patients with erysipelas or cellulitis admitted to the dermatology department of the university hospital Halle between January 2024 and January 2025. They had been managed according to an internal standard operation procedure for skin‐ and soft tissue infections.

**Results**: Of 76 erysipelas patients without other infections, 91.4% reported constitutional symptoms at or before erythema onset, among them 17 of 18 patients with recurrent erysipelas. In contrast, only 36.2% of 47 cellulitis patients experienced such symptoms, typically later and not before erythema. Of patients with erysipelas, 98.3% responded within 2 days to penicillin, including 21 patients who had experienced symptoms for already 4 to 10 days prior to therapy.

**Conclusions**: Our findings suggest that early constitutional symptoms and characteristic erythema are reliable indicators for erysipelas. Erysipelas do not tend to heal promptly and spontaneously but respond reliably to penicillin.

## INTRODUCTION

Erysipelas and uncomplicated or limited cellulitis (phlegmon)^1^ rank among the most common skin and soft tissue infections (SSTIs) worldwide.^2^, ^3^, ^4^ No precise registry data on their incidences are available. Given the inconsistent usage of definitions, international studies are comparable only to a limited extent. According to the German Federal Statistical Office, approximately 70,000 patients with the diagnosis of erysipelas were treated as inpatients in Germany in 2016.^5^

Despite their high incidence, these disease entities are not always differentiated sufficiently with regard to nomenclature, clinical signs, or management. However, their differentiation is important for therapy: Usually, erysipelas can be treated adequately with penicillin alone, while cellulitis requires at least a penicillinase‐resistant narrow‐spectrum beta‐lactam antibiotic and in some cases even a broad‐spectrum antibiotic.^1^, ^6^ Although these beta‐lactam antibiotics are generally well tolerated, they have a slightly higher risk of toxicity than penicillin.^7^, ^8^, ^9^ Moreover, penicillin is less likely to alter the microbiome.^10^ Due to its very narrow spectrum, it will also select fewer resistant pathogens than an antibiotic with a broader spectrum.^11^

Classic erysipelas is considered an acute bacterial, clinically non‐purulent infection of the dermis caused by beta‐hemolytic streptococci.^1^, ^12^, ^13^ Although their detection by culture from tissue samples or swabs is usually not successful (summarized in^13^ and^1^, as well as a partial result from an ongoing investigation [Sunderkötter, Becker, et al., unpublished]), the response to penicillin shown in case series is considered to present an indirect evidence for streptococci as causative pathogen even without their detection.^14^

In contrast, uncomplicated cellulitis (phlegmon) is defined as an infection of the dermis with very little or no pus production, usually caused by *Staphylococcus (S.) aureus*, which may extend to the subcutaneous tissue.^1^ It presents neither erysipelas caused by β‐hemolytic streptococci nor a complicated purulent necrotizing soft tissue infection extending to the fascia. While tentatively identified as “limited cellulitis” in the first German S2k guideline on SSTIs ^1,^ ^15^, they are defined as “uncomplicated cellulitis” in the updated version (not yet released), given that this term allows better differentiation from the severe cases, now called “complicated” cellulitis. ^15^ The latter are defined using the criteria for “complicated skin and soft tissue infections” as originally established by the FDA for clinical trials.^16^

Erysipelas and cellulitis are differentiated by their clinical features.^1^, ^17^ Uncomplicated cellulitis is characterized by a warm, pasty swelling often tender to palpation with dark or livid erythema that is less shiny and less sharply demarcated than in classic erysipelas. From experience, it usually develops around an ulcer or a wound as port of entry.^1^ In contrast, erysipelas presents clinically as acute, warm, more or less painful, bright red erythema with a shiny surface, relatively well‐defined borders, sometimes with arched extensions.^1^ Based on case reports and expert opinion, “constitutional symptoms” are often mentioned as a potentially diagnostic sign in addition to these clinical and morphological criteria.^1^, ^18^ These symptoms include nausea, shivers (rarely, chills), and fever as signs of a systemic inflammatory reaction. To date, however, no study or larger case series has provided evidence for this criterion. While fever and chills have been used as inclusion criteria in e.g. some older French studies,^13^, ^18^, ^19^ they have not been explicitly discussed^20^ or mentioned in other studies specifically addressing the etiology or characteristics of erysipelas.^14^, ^21^

Given that in our hospital patients with soft tissue infections for the last 3 years have been asked routinely about constitutional symptoms, and that clinical data have been recorded according to a standard operating procedure, we wanted to analyze the following aspects retrospectively based on patient records: *(1)* How often were constitutional symptoms, i.e. fever (≥ 38 °C), chills or shivers, or feeling of illness reported in patients diagnosed with erysipelas or uncomplicated (limited) cellulitis according to clinical criteria, *(2)* how often did patients with erysipelas respond to therapy with penicillin(as recommended in guidelines), and *(3)* were additional conspicuous features observed that might facilitate differentiation between erysipelas and uncomplicated cellulitis.

## PATIENTS AND METHODS

We included all patients of the Department of Dermatology of the University Hospital Halle who received inpatient treatment because of erysipelas or uncomplicated (limited) cellulitis from January 2024 to January 2025 in our retrospective cohort study. They were identified by their respective discharge diagnosis. Patients with complicated cellulitis (complicated skin and soft tissue infections, SSTIs), necrotizing SSTIs, or abscesses were excluded.

The diagnosis of erysipelas was made clinically in case of an erythema with bright red (instead of livid) color, rather shiny surface, and relatively well‐defined borders without underlying pasty swelling or abscess formation.

The diagnosis of cellulitis was made in case of livid‐red erythema with poorly defined borders and underlying edematous or pasty swelling. Constitutional symptoms or fever did not exclude the diagnosis.

The presence or absence of symptoms relevant for the diagnosis was documented according to the standard operating procedure of our department. In addition, the following parameters were recorded routinely:

- potential ports of entry,
- level of pain,
- history of constitutional symptoms, such as fever, shivers or chills, malaise, fatigue, and nausea (Table 1). To this end, all patients with soft tissue infections were asked about respective symptoms according to internal work instruction (standard operating procedure) (for example: “Shortly before or after manifestation of the redness, did you feel like having caught a cold, did you experience shivers or chills or did you have fever, or did you feel fatigued?”),
- history concerning the duration of symptoms before presentation and before onset of treatment,
- chronic lymphedema or lipolymphedema at the site of infection,
- inflammation parameters: C‐reactive protein (CRP) and blood count,
- additional acute infections being present at the time of admission causing constitutional symptoms or requiring consideration when selecting the antibiotic therapy.

**TABLE 1:** Constitutional symptoms as inquired (multiple answers possible).

Reported constitutional symptoms in erysipelas (n = 76)	
Fever	39 (51.3%)
Shivers (partly reported as “chills” by patients)	32 (42.1%)
Night sweat	2 (2.6%)
Fatigue and decreased general condition	34 (44.7%)
Nausea/vomiting	9 (11.8%)
Arthralgia, aching limbs	2 (2.6%)

Penicillin G was used for treating erysipelas; the standard dose was 10 million I.U. three times daily. In case of a history of confirmed or suspected penicillin allergy, cefazolin 2 g three times daily with emergency preparedness (to guarantee first aid in case of anaphylaxis) was the intended first alternative.^22^ Patients diagnosed with cellulitis received parenteral therapy with cefazolin (1‐2 g three times daily) in accordance with the guidelines. In case of extensive infection or of respective diagnostic/laboratory anomalies, ampicillin/sulbactam (2 g/1 g three times daily) or – if there were additional infections such as complicated urinary tract infections – cefuroxime (1.5 g three times daily) was administered.

Response to therapy was documented according to an internal standard operating procedure, if the following criteria were met:

- In erysipelas: Appearance of fine wrinkles on the erythematous skin (evidence of reduction of subepidermal edema) and/or decrease of the erythematous area, or fading of erythema.
- In cellulitis: Decrease of the erythematous area and/or reduction of the pasty swelling underlying the erythema, or fading of erythema.
- In addition, for both entities: Improvement of potential constitutional symptoms and pain, reduction of CRP levels (by at least 25% after 3 days) or leukocyte count (after 24 hours at the earliest; absolute numbers, no percentage values).

### Ethics vote

The retrospective analysis of the data was conducted with consent of the local ethics committee (Medical Faculty of the Martin Luther University Halle‐Wittenberg) (0205‐022).

## RESULTS

Between January 01, 2024, and January 31, 2025, altogether 123 patients with the discharge diagnosis of erysipelas (n = 76) or cellulitis (n = 47) were identified in the archive of our dermatology department. In five cases, the initial diagnosis of “erysipelas” was corrected to “cellulitis” (discharge diagnosis) during hospitalization based on the presence of a pronounced or pasty local swelling (all of these patients had constitutional symptoms); accordingly, these patients were classified as cellulitis in this analysis (Tables S1, S2 in the Online Supplement).

### Clinical appearance

**FIGURE 1:**
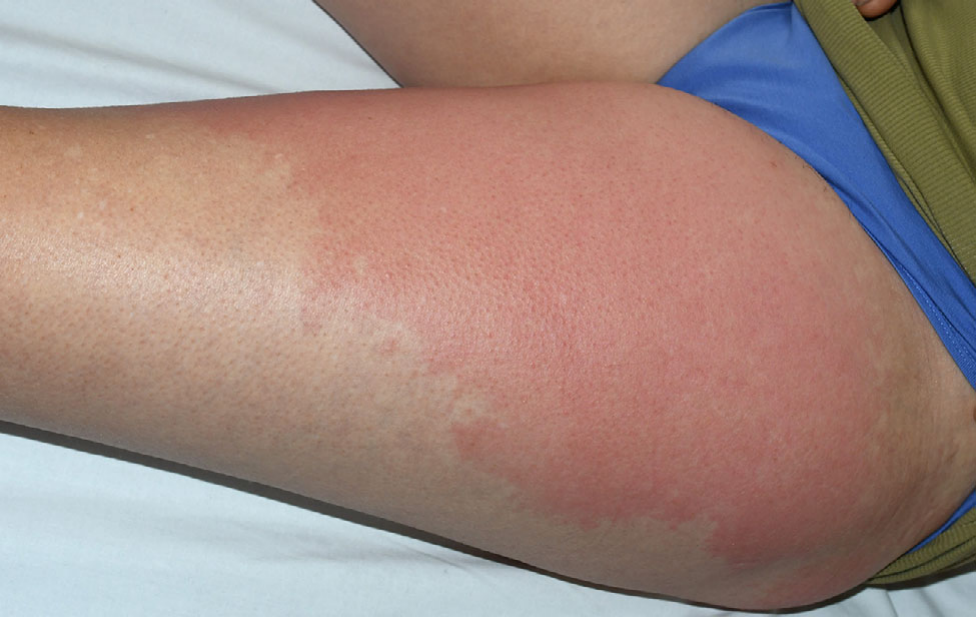
Classic erysipelas on the thigh with arched (flame‐shaped) extensions.

**FIGURE 2:**
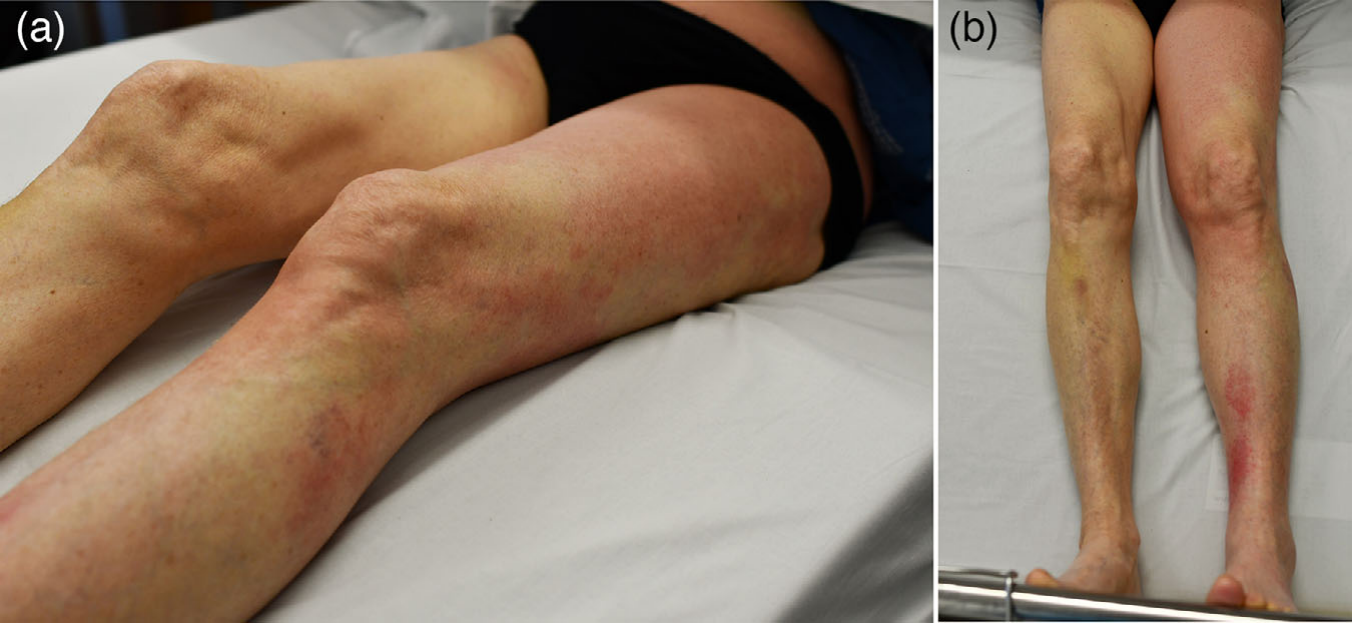
Erysipelas on the leg with discontinuous, island‐like distribution of erythema in chronic lymphedema. (a) Lateral view. (b) frontal view which shows the lympedema of the left in contrast to the right leg.

**FIGURE 3:**
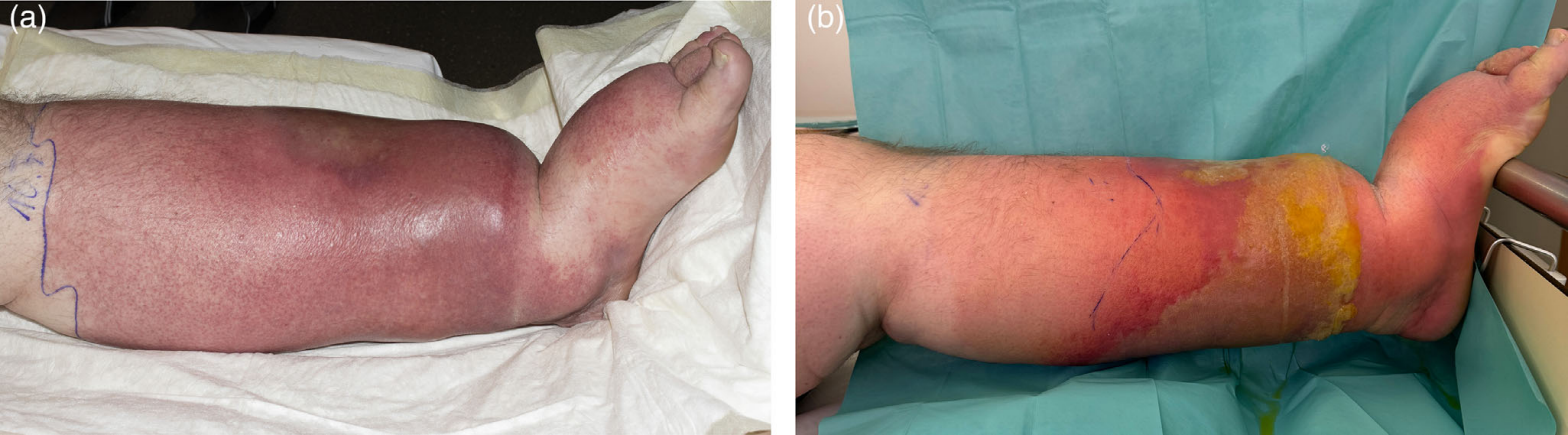
Erysipelas in chronic lymphedema with formation of central bulla. (a) Day 2 with incipient bulla formation. (b) Day 6, the bulla has filled extensively with a serous, yellowish, but not purulent exudate, while erythema has partially faded.

In the patients with erysipelas, a continuous bright red or fiery red erythema was described in 70 cases (92.1%) (Figure 1). In addition, livid portions within the bright red erythema were described in six patients (7.9%). In five of these cases, the erysipelas had developed on chronic lymphedema, and here the otherwise continuous erythema was partly broken up into individual island‐like erythematous lesions, often only connected via a narrow band (Figure 2a, b). On nine of the other erysipelas, bullae filled with serous exudate or hemorrhagic bullae (n = 6) and/or bleedings (n = 4) were described additionally (in some cases, the yellowish serous exudate became more marked while the erythema had already faded in response to therapy) (Figure 3a, b).

**FIGURE 4:**
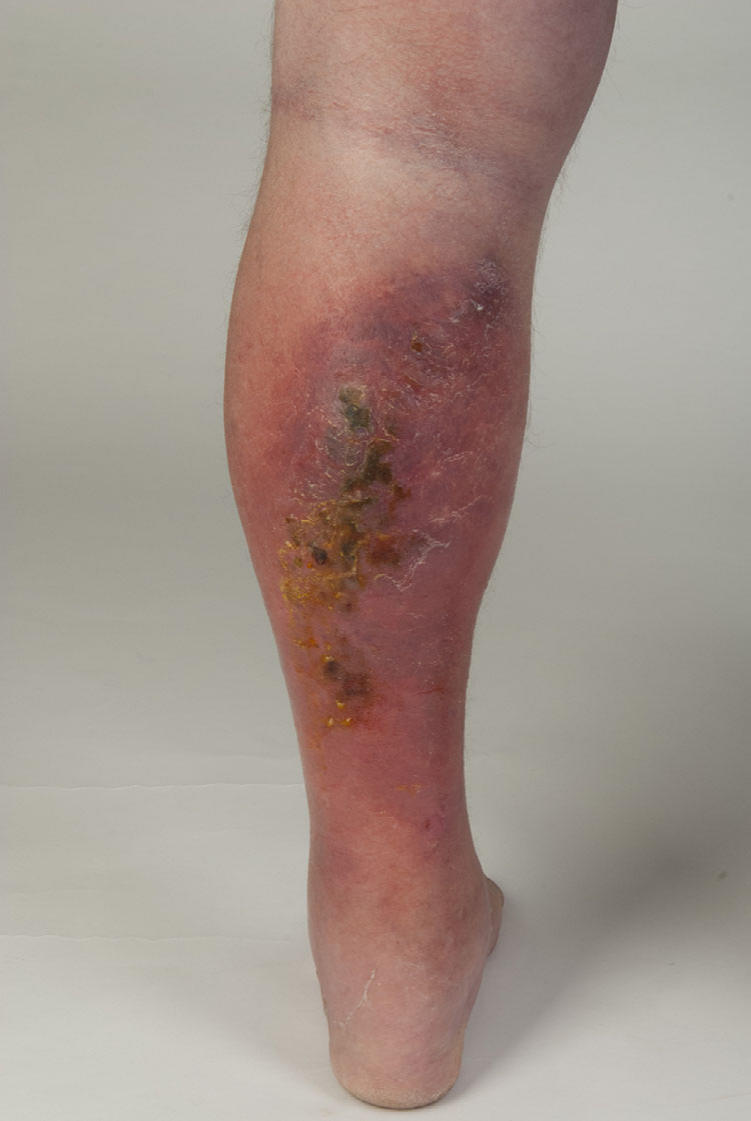
Uncomplicated cellulitis of the lower leg with less well‐defined border and predominantly livid‐red color.

In patients diagnosed with uncomplicated or limited cellulitis, a red erythema on a more or less pronounced pasty swelling was described in 33 of 47 cases (70.2%), while a red‐livid erythema was described in 13 cases (27.7) (Figure 4) and an additional portion with bright red color was described in one case only (2.1%). These erythematous lesions were characterized by poorly defined borders. Most erysipelas and cellulitis lesions were localized on the lower legs (Table 2).

**TABLE 2:** Skin areas involved in erysipelas and uncomplicated cellulitis.

Localization	Erysipelas (n = 76)	Cellulitis (n = 47)
Whole leg	5 (6.6%)	1 (2.1%)
Thigh/groin	2 (2.6%)	5 (10.6%)
Lower leg	53 (69.7%)	18 (38.3%)
Foot	2 (2.6%)	2 (4.3%)
Face	14 (22.4%)	9 (19.1%)
Arm	2 (2.6%)	4 (8.5%)
Hand	0	3 (6.4%)
Breast	1 (1.3%)	0
Buttocks/back	0	2 (4.3%)
Abdomen	0	1 (2.1%)
Axilla in case of lymphocele	0	1 (2.1%)
Scalp	0	1 (2.1%)

### Constitutional symptoms

Of the patients with erysipelas, 92.1% (70 of 76) reported that they had experienced constitutional symptoms in the form of fever, chills, shivers, and feeling fatigued or ill at or before detection of the erythema (Table 1). Among this group, 17 of 18 patients had recurrent erysipelas (14 with fever or chills [2 despite antibiotic prophylaxis], 3 with fatigue). Nine patients also reported nausea and vomiting (Table 1). Almost all patients (n = 66) experienced and described these symptoms as significant and impairing. In six patients, an additional infection was identified after admission. Four patients had an otherwise asymptomatic urinary tract infection and two patients had bronchopneumonia. Although the constitutional symptoms coincided with the occurrence of erythema and were, therefore, uncharacteristic for the frequently asymptomatic urinary tract infections, these cases were not included in calculating the percentage of erysipelas with constitutional symptoms. Accordingly, the percentage was 91.4% (64 of 70).

Of the six patients without documented constitutional symptoms, two were on permanent therapy with metamizole (an antipyretic), one patient was already in a markedly reduced general condition after radiotherapy, and two patients received already oral antibiotics (the latter, however, did not always prevent constitutional symptoms, because five of seven other patients reported them despite already ongoing oral treatment with antibiotics). The information that constitutional symptoms are sometimes observed already 1–2 days before the erythema, had motivated us to explicitly inquire about this factor from each patient and document the result since October 2024. Based on these data, in twelve of 20 cases with erysipelas, chills or fever and fatigue were experienced already prior to (n = 7) or in conjunction with (n = 5) an erythema which at that point affected only a small area. In some patients, the constitutional symptoms had already declined to a greater or lesser extent at the time of presentation.

In contrast, only 36.2% (n = 17) of the patients with uncomplicated cellulitis reported constitutional symptoms, mostly fever and chills; two of these patients had received antibiotic treatment previously. In seven cases the symptoms were noticed concomitantly with the erythema, in five cases they appeared after redness or swelling had progressed, and in five cases no information was available. In no case these symptoms were documented to have occurred before the onset of skin changes, although these patients, too, had been asked explicitly about this aspect since October. In patients without constitutional symptoms, no long‐term administration of anti‐inflammatory drugs had been documented.

### Pain

Pain was reported in 47 patients with erysipelas (59.5%), the other 34 patients (43%) had denied having pain. Three patients experienced strong localized pain increasing upon pressure in the erythematous area. While thrombosis could be excluded in each case, marked focal edema restricted to single connective tissue septa at the point of maximal pain was identified in ultrasound scans. In cellulitis, pain was affirmed by 27 patients (57.4%) and denied by 18 patients (38.3%), while no information was available for two patients.

### Potential ports of entry

In erysipelas, maceration or erosion (mostly, interdigital mycosis) were described in 50 cases (68.5%), while in many other cases no and in only few case a larger port of entry had been observed (Table 3). It was not always possible to detect bacteria from corresponding swabs, but when bacterial cultures turned positive they grew *S. aureus* or streptococci (unpublished data).

**TABLE 3:** Potential ports of entry in erysipelas and uncomplicated cellulitis.

Superficial defects		
	*Erysipelas*	*Cellulitis*
Maceration, mycosis, erosion	50	7
No port of entry evident	21	4
Impetiginized erosions	0	6
Folliculitis	0	2
Rhagades	0	1
Erosion in calcinosis cutis	0	1
*Total*	71 (93.4%)	21 (44.7%)

Deep defects		
	*Erysipelas*	*Cellulitis*
Ulcer	3 (shallow ulcers)	9 (including 1 pressure ulcer)
Fresh tattoo	1	0
Surgical wound	1	5
(Incised) wound	0	3
Abscess	0	4
Manipulated cyst	0	2
Furuncle	0	1
Lymphocele, status after puncture	0	1
Bursitis	0	1
*Total*	5 (6.6%)	26 (56.5%)

In cellulitis, ulcers (19.1%), abscesses (14.9%), or surgical wounds were often documented as port of entry (Table 3). Various gram‐negative bacteria and almost always *S. aureus* could be cultivated from swabs (unpublished data).

### Lab parameters

At admission, patients diagnosed with erysipelas had mean CRP levels of 81.49 mg/dl (range 3–219.6 mg/dl; standard deviation [SD] 77.2) and mean leukocyte counts of 11.74 × 10^9^/l (range 4.1–25 × 10^9^/l; SD 5.34). Patients with cellulitis had mean CRP levels of 95.88 mg/dl (range 2.8–451 mg/dl; SD 110.26) and mean leukocyte counts of 12.73 × 10^9^/l (range 5.1–43.8 × 10^9^/l; SD 5.80). Due to the wide variation, interpretation of these figures is limited. There was no significant difference between erysipelas and uncomplicated cellulitis with respect to CRP levels or leukocyte counts.

### Therapy and response

Sixty of the 76 patients with erysipelas were treated with penicillin; of these, two patients with hemorrhagic bullae received also short‐term treatment with clindamycin to inhibit toxin synthesis. Because of a reported history of a drug reaction or an allergy to penicillin or a respective note in the allergy card, six patients were treated with cefazolin under emergency preparedness (to guarantee first aid in case of anaphylaxis) according to the internal standard operating procedure. Three patients with suspected additional staphylococcal (super‐)infection also received cefazolin. The six patients with a second infection (pneumonia or urinary tract infection) were treated with ampicillin/sulbactam, cefuroxime, or ceftriaxone (all IV) after that diagnosis was made. One patient developed aspiration pneumonia later during hospitalization and was then switched to ampicillin/sulbactam. Five patients had been given oral antibiotics before, but had not responded to this treatment (2 x penicillin V, 1 x penicillin V and azithromycin, 1 x clindamycin, and 1 x an antibiotic that could not be remembered or identified).

Of the 60 patients treated with penicillin, 59 patients (98.3%) responded within 2 days, revealing fine wrinkling of the skin, followed by fading of redness and then reduction of the erythematous area accompanied by an improvement of still existing constitutional symptoms. In one case, the antibiotic therapy was complemented by clindamycin after 3 days because of an apparently insufficient clinical response and the absent decrease of CRP levels. This resulted in a response.

In patients treated with penicillin, eleven had experienced symptoms for more than 6 days and ten had experienced them for at least 4 to 6 days before initiation of therapy. Nevertheless, no concomitant infections were identified in these patients. All of them responded within 2 days to the antibiotic therapy.

Of the 47 patients diagnosed with cellulitis, 36 were treated with cefazolin, three with ampicillin/sulbactam, two with clindamycin, and one with cefuroxime. Those patients diagnosed with erysipelas at admission who had not responded adequately to the initial treatment with penicillin received cefazolin (n = 3) or ampicillin/sulbactam (n = 2). While the antibiotic was switched in six cases from cefazolin to ampicillin/sulbactam after 2 days due to an inadequate response, the remaining cases responded to the therapy within 2–3 days with reduction of the erythematous area and the pasty swelling underlying the erythema as well as with fading of redness. Only two patients had already been treated with oral antibiotics in an outpatient setting (penicillin V and amoxicillin/clavulanic acid), which had not resulted in relevant improvement of symptoms.

## DISCUSSION

In the analyzed cohort, we found that *(1)* more than 90% of the patients with erysipelas had constitutional symptoms, always at onset of infection, *(2)* in uncomplicated cellulitis, however, constitutional symptoms were reported only in 36% of the cases and sometimes only in the course of disease, and *(3)* therapy with penicillin was effective in erysipelas, when these symptoms were present. Given these data, it might be concluded that the presence of constitutional symptoms at disease onset in addition to the characteristic erythema is a crucial criterion for the clinical diagnosis of erysipelas.

The constitutional symptoms appearednot before emergence of the erythema also in all patients who were additionally diagnosed with a pre‐existing infection after admission, usually an otherwise asymptomatic urinary tract infection. In the six patients without documented constitutional symptoms, therapy with metamizole or antibiotics might have suppressed the constitutional symptoms or prevented them at an early stage. In another patient, a weakened condition after radiation was documented in the patient record. Accordingly, in five of the six patients, there was at least one potential reason why the constitutional symptoms were not reported or observed. While the constitutional symptoms may also include nausea and vomiting, these seem to occur only in association with one of the other symptoms mentioned above.

In uncomplicated cellulitis, however, constitutional symptoms occurred not only significantly less often (36.2%), but in some cases also explicitly only during the course of infection; in our cohort, no case was reported where constitutional symptoms occurred before the erythema was noticed, in contrast to several cases with erysipelas.

Although the clinical features of erysipelas with their bright red, often shiny and arched erythema are often clearly distinguishable from the livid‐red, pasty swelling of uncomplicated cellulitis, the addition of constitutional symptoms as an diagnostic criterion would provide additional security in making the diagnosis. Even more so when they occur at disease onset. Therefore, based on our experience, it may be useful to ask patients with soft tissue infection not only about current presence of constitutional symptoms, but also explicitly whether such constitutional symptoms were noticed already around the onset of disease. Due to their early and apparently sometimes transient manifestation, they may not be actively reported or brought into context with the subsequently observed erythema. If the respective question would not have been part of our routine assessment of patients with soft tissue infections, these initial constitutional symptoms likely would have been documented less often in the patient record. These circumstances may explain why constitutional symptoms were not always used as specific criterion.^20^ Fever and chills were, for example, mentioned as inclusion criteria in French studies on *erysipelas/cellulitis*, but apart from that they were not specifically evaluated or discussed.^13^, ^19^

To date, no corresponding studies are available on the potential causes for these early constitutional symptoms. However, well‐conceived systematic studies on acute streptococcal pharyngitis provide some evidence: only when challenge with group A streptococci resulted in a manifest infection, a marked increase of, among other factors, interleukin‐1 receptor antagonists (IL‐1Ra) and interleukin‐18 (IL‐18) was measured.^23^ These cytokines contribute directly or indirectly to fever and other inflammatory signs and are induced by several of the 13 superantigens and additional virulence factors of streptococci.^24^

Another striking difference between erysipelas and uncomplicated cellulitis was the port of entry: In cellulitis, usually a larger lesion which involved deeper skin layers was documented, whereas in erysipelas often no clear port of entry or only minor erosions (usually macerations in association with interdigital mycosis) were described.

In our cohort, CRP levels and leukocyte counts were frequently elevated to a different extent in both erysipelas and cellulitis. In contrast to another retrospective study (with participation of C.S.)^25^, we could not identify significantly higher values in erysipelas. However, the significance of our results is limited due to the large variance. This may be caused by the fact that the patients presented at different disease stages and sometimes only after the symptoms had already been present for a certain period.

The appearance of bright red erythema with, in part, sharply defined, also arched borders was a differentiating inclusion criterion. Strikingly, the otherwise homogenous redness appeared in some cases not evenly bright red, but was broken up into island‐like lesions, usually when erysipelas developed on chronic lymphedema. It was also noticeable that in a few cases single or several small serous bullae were present at admission which occasionally filled up with yellowish serous exudate at a later stage when the erythema had already responded to therapy. Accordingly, this course with formation of bulging bullae is not a sign of disease progression; the serous content must not be misinterpreted as pus.

Pronounced acute edemas underlying the erythema are untypical for erysipelas – except when they manifest in the face or the genital region, where the loose structure of connective tissue facilitates the accumulation of interstitial fluid and formation of edema. In these cases, differential diagnosis from cellulitis is challenging. Accordingly, the absence of initial constitutional symptoms could become the decisive criterion for a diagnosis of cellulitis and against a diagnosis of erysipelas. Based on our results, it would indeed be justified to consider the presence of shivers, fever, chills, and/or fatigue at onset of infection as an almost essential, albeit insufficient criterion for the diagnosis of erysipelas. This hypothesis could be confirmed by a prospective, if possible multicentric study that would also record more precisely and widely *(1)* how often constitutional symptoms are observed already before or immediately at manifestation of the erythema, *(2)* how long they persist or how often they resolve before starting the therapy, and *(3)* whether they are associated with increased levels of IL‐1Ra or IL‐18, if applicable. Including cases with uncomplicated cellulitis would also allow for the prospective analysis of the extent to which these symptoms are less common at onset of cellulitis and how often they develop only during the disease.

According to a meta‐analysis, broad‐spectrum antibiotics with corresponding adverse consequences are used too often for the therapy of erysipelas and uncomplicated cellulitis up to this day. This weighs heavily, given the relatively frequent occurence of these soft tissue infections in clinics or hospitals of dermatology, general medicine and pediatrics.^26^ Instead, antibiotics with narrow efficacy spectrum that have been available for years are sufficient and still recommended even without recent randomized studies.^6^, ^15^ For erysipelas, penicillin (for example, benzylpenicillin IV 3 × 10 million IU/day for 7 days) is considered to be the drug of first choice, although erysipelas and uncomplicated cellulitis were not always clearly distinguished in the respective case series.^12^, ^27^ With a response rate of 98.3%, our retrospective study confirms the efficacy of penicillin in erysipelas. The additional administration of clindamycin in individual severe cases (n = 2) with hemorrhagic bullae or seropapules is based on expert recommendation and is derived from one study showing that inhibition of bacterial protein synthesis and thus toxin production by administration of clindamycin for three days reduced the mortality in toxin‐mediated, necrotizing soft tissue infections.^28^

Our observations concerning the necessitiy for treatment showed that several patients had an erythema already for several days before seeking medical advice, but then responded promptly to our therapy within two days. This indicates that erysipelas – despite the frequently unsuccessful pathogen detection – shows no spontaneous tendency to heal quickly, and therefore requires targeted therapy, preferably with penicillin.

Of the patients with cellulitis, 83.3% responded to therapy with penicillinase‐resistant narrow‐spectrum beta‐lactam antibiotic (cefazolin); in the remaining cases, a response to the drugs recommended as next option in the guidelines was observed.^6^, ^15^

Although these beta‐lactam antibiotics are usually well tolerated, they have a higher risk of adverse effects than penicillin.^7^ These include, for example, liver injury caused by a metabolite of flucloxacillin,^29^ crystalluria and kidney failure on high‐dose parenteral administration of ampicillin or amoxicillin^30^, and hepatotoxicity associated with clavulanic acid. In direct comparison with penicillin, oxacillin, nafcillin^8^ and – to a somewhat lesser extent – also cefazolin^9^ showed more adverse events. Therefore, they are not recommended as substances of first choice for treating erysipelas.^15^ Our retrospective analysis also suggests that no other beta‐lactam antibiotic than penicillin is required for treating erysipelas.

Potential limitations of our findings result from the retrospective study design, the inclusion of inpatients only, the collection and documentation of parameters by different physicians of our dermatology department, and a potential bias concerning the importance of constitutional symptoms. However, the following findings argue against a major bias: Constitutional symptoms were also documented in some cases of uncomplicated cellulitis, the original diagnosis was corrected from erysipelas to cellulitis in five patients – despite reported constitutional symptoms, and the response rate to penicillin in all cases classified as erysipelas was almost 100%; given the penicillinase‐producing *S. aureus*, one would not expect this high response rate if cellulitis had erroneously been diagnosed as erysipelas.

## CONFLICT OF INTEREST STATEMENT

C.S. has received honoraria for lectures and advisory activities in connection with soft tissue infections from Bayer AG, Correvio, Infectopharm, GlaxoSmithKline, and Novartis. H.S. declares no conflict of interest.

## Supporting information



Supporting Information
