# Supplementary material for: Constitutional symptoms and response to Penicillin G in erysipelas and cellulitis – a monocentric, retrospective, explorative study
Source: J Dtsch Dermatol Ges. 2026 Jan 21;24(6):746–54. doi: 10.1111/ddg.15957 (PMC13238294; doi:10.1111/ddg.15957)
Supplement: Supplementary file 1 — Supporting Information [file DDG-24-746-s001.docx]

# Supplemetary Tabellen für Publikation Erysipel

| **Alter (Jahre)** | **Geschlecht** | **Chronisches Ödem** | **Atopische Dermatitis (bei Lokalisation im Gesicht)** | **Rezidiv** |
| --- | --- | --- | --- | --- |
| 23 | weiblich | nein |  | 6. Rezidiv |
| 87 | weiblich | Varikosis |  | 1. Rezidiv |
| 53 | weiblich | nein | nein | nein |
| 37 | weiblich |  | ja | nein |
| 92 | weiblich | Varikosis |  | nein |
| 59 | weiblich |  | nein | 1. Rezidiv |
| 77 | männlich | nein |  | nein |
| 40 | weiblich |  | ja | 1. Rezidiv |
| 57 | weiblich | chron. Lymphödem |  | nein |
| 82 | männlich | chron. Lymphödem |  | nein |
| 56 | männlich | Stauungsdermatitis |  | nein |
| 48 | weiblich | Lymphödem bei Z.n. LAE und Radiatio vor 7 Wochen |  | nein |
| 61 | weiblich | nein |  | nein |
| 85 | weiblich | chron. Lymphödem |  | 18. Rezidiv in 22 Jahren |
| 82 | männlich | Stammvarikosis |  | 1. Rezidiv |
| 83 | männlich | Phlebolymphödem |  | 1. Rezidiv |
| 83 | männlich | nein |  | nein |
| 94 | weiblich | nein |  | nein |
| 58 | männlich | nein |  | nein |
| 84 | weiblich | Stammvarikosis mit Stauungsdermatitis |  | nein |
| 98 | weiblich | Stauungsdermatitis |  | nein |
| 51 | männlich |  |  | nein |
| 64 | weiblich | chron. Lymphödem |  | nein |
| 44 | weiblich | nein |  | nein |
| 72 | weiblich | chron. Lymphödem |  | nein |
| 75 | weiblich | nein |  | nein |
| 62 | weiblich | chron. Lymphödem |  | 1. Rezidiv |
| 83 | männlich | Stauungsdermatitis |  | 2. Rezidiv |
| 43 | männlich |  | ja | nein |
| 60 | weiblich |  | nein | 3. Rezidiv |
| 83 | männlich | Stauungsdermatitis |  | 2. Rezidiv |
| 33 | männlich | chron. Lymphödem |  | nein |
| 69 | männlich | nein |  | nein |
| 51 | männlich | nein |  | nein |
| 81 | männlich | chron. Lymphödem |  | 3. Rezidiv |
| 74 | weiblich | nein |  | nein |
| 40 | männlich | chron. Lymphödem |  | nein |
| 82 | männlich | nein |  | nein |
| 46 | männlich | nein |  | nein |
| 80 | männlich | nein |  | nein |
| 63 | weiblich | nein |  | 1. Rezidiv |
| 88 | männlich | chron. Lymphödem |  | nein |
| 34 | männlich | nein |  | nein |
| 55 | weiblich | Lipödem |  | nein |
| 60 | männlich | Stauungsdermatitis |  | 1. Rezidiv |
| 50 | männlich | nein |  | nein |
| 48 | männlich | nein |  | nein |
| 51 | männlich | nein |  | nein |
| 58 | männlich | nein |  | nein |
| 76 | weiblich | chron. Lymphödem |  | nein |
| 84 | männlich | chron. Lymphödem |  | 2. Rezidiv |
| 83 | weiblich | nein |  | nein |
| 79 | weiblich | chron. Lymphödem |  | nein |
| 54 | weiblich |  | nein | nein |
| 75 | weiblich | chron. Lymphödem |  | nein |
| 36 | männlich | nein |  | nein |
| 88 | weiblich |  | nein | nein |
| 65 | männlich | nein |  | nein |
| 60 | weiblich | nein |  | nein |
| 72 | weiblich |  | nein | nein |
| 61 | weiblich | nein |  | nein |
| 82 | männlich | nein |  | nein |
| 58 | weiblich |  | nein | nein |
| 87 | weiblich | chron. Lymphödem |  | nein |
| 85 | männlich | nein |  | nein |
| 59 | männlich |  | nein | nein |
| 85 | weiblich | nein |  | nein |
| 55 | männlich | nein |  | nein |
| 87 | weiblich | chron. Lymphödem |  | 1. Rezidiv |
| 34 | männlich | Varikosis bei CVI |  | nein |
| 66 | weiblich | Lipolymphödem |  | nein |
| 62 | weiblich | Lipolymphödem |  | nein |
| 57 | männlich | nein |  | nein |
| 87 | weiblich | nein |  | 1. Rezidiv |
| 46 | männlich |  | nein | nein |
| 49 | männlich | nein |  | nein |

**Tabelle 1:** Allgemeine Patientencharakteristika Erysipel

Table 1: Common patient characteristics erysipelas

*CVI = chronisch-venöse Insuffizienz; LAE = Lymphadenektomie*

| **Alter (Jahre)** | **Geschlecht** | **Chronisches Ödem** | **Atopische Dermatitis (bei Lokalisation im Gesicht)** | **Rezidiv** |
| --- | --- | --- | --- | --- |
| 57 | männlich | paVK Stadium IV bds. |  | nein |
| 81 | weiblich | nein |  | nein |
| 54 | männlich | nein |  | nein |
| 62 | weiblich | nein |  | nein |
| 80 | männlich | nein |  | nein |
| 63 | männlich | nein |  | nein |
| 96 | weiblich | nein |  | nein |
| 34 | männlich | nein |  | nein |
| 79 | weiblich | nein |  | nein |
| 86 | weiblich | nein |  | nein |
| 37 | weiblich | nein |  | nein |
| 59 | weiblich |  | nein | nein |
| 40 | männlich | - | - | ja |
| 55 | weiblich | - | nein | nein |
| 82 | weiblich |  | nein | nein |
| 67 | männlich | Lymphödem, Stauungsdermatitis, Dermatoliposklerose |  | nein |
| 68 | weiblich |  | nein | nein |
| 69 | weiblich | Chron. Lymphödem |  | nein |
| 82 | weiblich | - | - | nein |
| 84 | weiblich | - | - | nein |
| 27 | weiblich | - | - | nein |
| 20 | männlich | nein | - | nein |
| 59 | weiblich | - | nein | 1. Rezidiv |
| 66 | weiblich | nein |  | nein |
| 82 | weiblich | - | - | nein |
| 46 | männlich | Stauungsekzem bei Unterschenkelödemen bds. | - | nein |
| 50 | weiblich | Dermatoliposklerose, Calcinosis cutis | - | nein |
| 83 | weiblich | Chron. Lymphödem | - | nein |
| 38 | weiblich | - | - | nein |
| 69 | männlich | - | nein | nein |
| 72 | männlich | - | - | nein |
| 40 | männlich | nein | - | nein |
| 74 | weiblich | - | - | nein |
| 64 | weiblich | Chron. Lymphödem | - | nein |
| 24 | weiblich | nein | - | nein |
| 82 | männlich | - | - | nein |
| 65 | weiblich | nein | - | nein |
| 21 | männlich | nein | - | nein |
| 66 | männlich | nein | - | nein |
| 32 | männlich | nein | - | nein |
| 63 | weiblich | nein | - | nein |
| 82 | männlich | nein | - | nein |
| 75 | männlich | nein | - | nein |
| 72 | weiblich | - | - | nein |
| 78 | männlich | nein | - | nein |
| 84 | weiblich | nein | - | nein |
| 89 | weiblich | - | nein | nein |

**Tabelle 2**: Allgemeine Patientencharakteristika unkomplizierte Phlegmone

Table 2: Common patient characteristics uncomplicated Phlegmon

*paVK = periphere Arterielle Verschlusskrankheit; bds. = beidseits*
